# Draft Genome Sequence of the Gram-Positive Thermophilic Iron Reducer *Thermincola ferriacetica* Strain Z-0001^T^

**DOI:** 10.1128/genomeA.01072-15

**Published:** 2015-09-24

**Authors:** Bradley G. Lusk, Jonathan P. Badalamenti, Prathap Parameswaran, Daniel R. Bond, Cesar I. Torres

**Affiliations:** a Swette Center for Environmental Biotechnology, The Biodesign Institute at Arizona State University, Tempe, Arizona, USA; b Department of Microbiology and BioTechnology Institute, University of Minnesota-Twin Cities, Saint Paul, Minnesota, USA; c School for Engineering of Matter, Transport and Energy, Arizona State University, Tempe, Arizona, USA

## Abstract

A 3.19-Mbp draft genome of the Gram-positive thermophilic iron-reducing *Firmicutes* isolate from the *Peptococcaceae* family, *Thermincola ferriacetica* Z-0001, was assembled at ~100× coverage from 100-bp paired-end Illumina reads. The draft genome contains 3,274 predicted genes (3,187 protein coding genes) and putative multiheme *c*-type cytochromes.

## GENOME ANNOUNCEMENT

*Thermincola ferriacetica* strain Z-0001 (DSM 14005), first isolated from a terrestrial hydrothermal spring on Kunashir Island (Kurils) (1), is a Gram-positive, thermophilic (45°C to 70°C), spore-forming bacterium that is capable of dissimilatory metal reduction and anode respiration in a microbial electrochemical cell (MXC) (2–4) and is one of only a limited number of sequenced Gram-positive thermophilic bacteria that has been documented to perform extracellular electron transfer (EET) to insoluble metal substrates (5–7). Strain Z-0001 is capable of organotrophic growth with acetate and other organic compounds while reducing extracellular electron acceptors, including amorphous Fe(III)-hydroxide, magnetite, Mn(IV), anthraquinone-2,6-disulfonate (AQDS), and anodes in MXCs (1, 2, 4). Strain Z-0001 is also capable of chemolithoautotrophic growth, using molecular hydrogen as the electron donor and Fe(III) as the electron acceptor (1). In addition, strain Z-0001 is produces H_2_ and CO_2_ while using CO as its electron donor and acquiring its carbon from acetate (1).

Among Gram-positive bacteria, little is known regarding the mechanism for EET or how the peptidoglycan layer impacts this pathway (8–10). Direct contact-dependent electron transfer has been suggested in *Thermincola potens* JR (11) with genetic evidence for the presence of *c*-type cytochromes (12), proteins which are responsible for EET in other metal-reducing bacteria (13). In contrast to *T. potens*, *T. ferriacetica* strain Z-0001 has been suggested to transfer electrons long range via an extracellular matrix (4), suggesting it may encode additional electron transfer capabilities. *Thermincola ferriacetica* has been reported to produce current densities up to 10 A·m^−2^ despite having only half the cytochrome repertoire of *Geobacter sulfurreducens* (4, 14). Further genetic comparison of these strains could help elucidate the EET mechanism(s) of strain Z-0001.

The draft assembly presented here is from an axenic culture of electrode-grown *T. ferriacetica* strain Z-0001 cells in order to eliminate contamination by iron or anthraquinone 2,6,-disulfonate (AQDS). gDNA was collected and sequenced on an Illumina HiSeq 2000 lane, yielding >45 million 2- × 100- bp reads. Raw reads were trimmed (sliding window 3 until quality >28) and down-sampled to provide 100× coverage for assembly using the a5 pipeline (26 Mart 2013 release [15]). The 3,196,047-bp draft genome assembly yielded 53 contigs >500 bp with an *N*_50_ of 112112 bp, an *L*_50_ of 8, and a G+C content of 45.69%.

The draft assembly was annotated using the JGI IMG/ER pipeline, yielding 51 tRNAs, 3,274 predicted genes (3,187 predicted protein coding genes), and 35 *c*-type cytochromes with three or more heme (CXXCH)-binding motifs. BLASTN sequence analysis of its 16S rRNA gene revealed 99.9% (1,436/1,438 nt) identity with *T. potens* JR and 99.7% (1,399/1,403 nt) identity with *Thermincola carboxydophila* (5, 16). *T. ferriacetica* contains two multiheme *c*-type cytochromes and 429 genes that are not present in *T. potens*. However, based on an average nucleotide identity (ANI) of 98.3% between their genomes, these two organisms may be members of the same species (17).

### Nucleotide sequence accession numbers.

This whole-genome shotgun project for *T. ferriacetica* strain Z-0001 has been deposited at DDBJ/EMBL/GenBank under the accession number LGTE00000000. The version described in this paper is version LGTE01000000. The raw and adaptor trimmed Illumina reads were submitted to SRA under accession number SRX1100231.
